# A global land cover training dataset from 1984 to 2020

**DOI:** 10.1038/s41597-023-02798-5

**Published:** 2023-12-07

**Authors:** Radost Stanimirova, Katelyn Tarrio, Konrad Turlej, Kristina McAvoy, Sophia Stonebrook, Kai-Ting Hu, Paulo Arévalo, Eric L. Bullock, Yingtong Zhang, Curtis E. Woodcock, Pontus Olofsson, Zhe Zhu, Christopher P. Barber, Carlos M. Souza, Shijuan Chen, Jonathan A. Wang, Foster Mensah, Marco Calderón-Loor, Michalis Hadjikakou, Brett A. Bryan, Jordan Graesser, Dereje L. Beyene, Brian Mutasha, Sylvester Siame, Abel Siampale, Mark A. Friedl

**Affiliations:** 1 Department of Earth and Environment, Boston University, 685 Commonwealth Avenue, Boston, MA 02215 USA; 2 Department of Geosciences and Natural Resource Management (IGN), University of Copenhagen, DK-1350 København K, Denmark; 3 NASA Marshall Space Flight Center, Huntsville, AL 35808 USA; 4 Department of Natural Resources and the Environment, University of Connecticut, Storrs, CT 06269 USA; 5 U.S. Geological Survey (USGS), Earth Resources Observation and Science (EROS) Center, Sioux Falls, SD 57198 USA; 6 Imazon—Amazonia People and Environment Institute, Belém, Brazil; 7 Yale School of the Environment, Yale University, New Haven, CT 06511 USA; 8 School of Biological Sciences, University of Utah, Salt Lake, UT 84112 USA; 9 Center for Remote Sensing and Geographic Information Services, University of Ghana, Accra, Ghana; 10 School of Life and Environmental Sciences, Deakin University, Melbourne, Australia; 11 Albo Climate, Ehad Ha’am, 9 Tel Aviv, Israel; 12 Grupo de Investigación de Biodiversidad, Medio Ambiente y Salud–BIOMAS, Universidad de las Américas (UDLA), Quito, Ecuador; 13 Indigo Ag, 500 Rutherford Avenue, Boston, MA 02129 USA; 14 REDD+ Coordination Unit, Oromia Environmental Protection Authority, Addis Ababa, Ethiopia; 15 Forestry Department Headquarters, Ministry of Green Economy and Environment, Lusaka, Zambia

**Keywords:** Biogeography, Ecosystem ecology

## Abstract

State-of-the-art cloud computing platforms such as Google Earth Engine (GEE) enable regional-to-global land cover and land cover change mapping with machine learning algorithms. However, collection of high-quality training data, which is necessary for accurate land cover mapping, remains costly and labor-intensive. To address this need, we created a global database of nearly 2 million training units spanning the period from 1984 to 2020 for seven primary and nine secondary land cover classes. Our training data collection approach leveraged GEE and machine learning algorithms to ensure data quality and biogeographic representation. We sampled the spectral-temporal feature space from Landsat imagery to efficiently allocate training data across global ecoregions and incorporated publicly available and collaborator-provided datasets to our database. To reflect the underlying regional class distribution and post-disturbance landscapes, we strategically augmented the database. We used a machine learning-based cross-validation procedure to remove potentially mis-labeled training units. Our training database is relevant for a wide array of studies such as land cover change, agriculture, forestry, hydrology, urban development, among many others.

## Background & Summary

The accuracy of land cover and land cover change maps derived from remote sensing data depends on training sample size and quality – two key considerations in planning and conducting supervised classification^1,2^. The amount of training data required can vary depending on the classification algorithm, the number of input variables, and the size and spatial variability of the mapped area^3–5^. For example, while most supervised classification algorithms (e.g., Random Forest) require a large training dataset of ‘pure’ class examples, some classification algorithms (e.g., Support Vector Machines) require smaller sets of mixed class samples for accurate land cover mapping^2–4,6–8^. Neural networks require a larger high-quality training set relative to other machine learning classification models^2,9^. Despite these differences, there is consensus that (1) large and accurate training datasets are generally preferable, and (2) classification accuracy increases with increasing training dataset size^2,5,6,10–13^.

As the global impact of climate change and anthropogenic activity has increased in recent decades, so has the need for high-quality maps of global land cover and land cover change. These maps require comprehensive, global, and high-quality land cover training datasets that are adaptable to the needs of a wide range of end users depending on the region of interest and the classification algorithm used. Currently, only a handful of continental-to-global training^14–18^ and validation^19,20^ datasets are publicly available. Several large-scale benchmark remote sensing datasets, designed to support the development of deep learning algorithms targeting specific applications, are publicly available (e.g., SpaceNet^21^, BigEarthNet^22^, and DeepSat^23^). While these datasets are valuable resources, the data collection efforts that produced them were uncoordinated and not standardized, largely because community-wide best practices for training data collection are not well established (although, see^2^). As a result, most of these datasets are limited by their geographic coverage, spatial resolution, observation density, time span, or quality.

The goal of the Global Land Cover Estimation (GLanCE) project is to provide high-quality long-term records of land cover and land cover change at 30 m spatial resolution for the 21st century using the Landsat archive^24^. As part of the GLanCE project, we present a new land cover training database that is designed to address the limitations outlined above. In creating this database, we aim to provide global coverage, ensure accuracy of land cover labels at 30 m spatial resolution, cover nearly four decades, and produce a geographically dense dataset. Our training data collection and curation approach leverages relatively recent technological advances such as cloud computing (e.g., Google Earth Engine (GEE)) and machine learning algorithms (e.g., Random Forest, k-means clustering etc.) to enforce data quality and ecological representation. Specifically, we implement an iterative quality assessment procedure that relies on expert review and a machine learning-based cross-validation procedure to remove poorly labeled training data.

Given the global scope of GLanCE, combined with the time and resource-intensive nature of training data collection, it was necessary to supplement the GLanCE data collection with external datasets and map products. Specifically, we harmonized seven publicly available land cover training datasets to be consistent with GLanCE data and combined them into a global database^14,16–20,25^. Similarly, we harmonized and integrated several collaborator-provided datasets^26–28^, along with datasets collected by Boston University team members for various other projects^29–34^. Lastly, following numerous recent studies (e.g.,^3,5,35,36^), we sampled existing land cover map products (i.e.,^18,37,38^) to fill geographic and thematic gaps in the dataset.

The objective of this paper is to describe the GLanCE training dataset, which is available to the public for use in regional-to-global land cover and land cover change studies. The dataset is global, medium spatial resolution (30 m), designed to be geographically and spectrally representative of all global ecoregions^39^, and spans the time period between 1984 and 2020. It provides a harmonized, standardized, and comprehensive database with up to 23 land cover characteristics per training unit. To support land cover change mapping, the dataset includes up to 36 years (in select regions of the world) and, notably, includes information about abrupt and gradual land cover change processes. It is designed to be global in scope but can be sub-sampled and adapted depending on the study region of interest, the classification algorithm used, and the classification legend desired (e.g., broad classes, land use, leaf type and phenology, etc.).

## Methods

### Training data collection

**Fig. 1:**
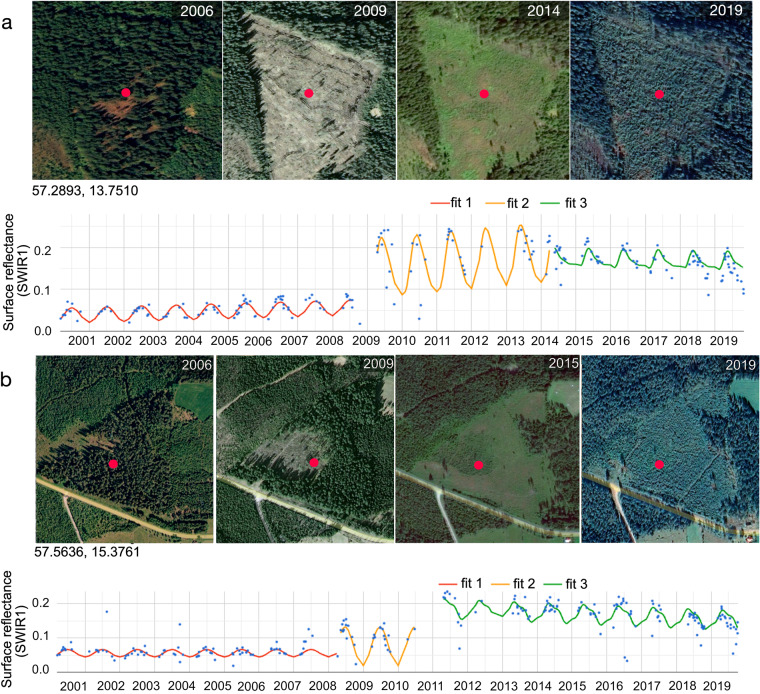
Continuous Change Detection and Classification (CCDC) model fits for two pixels in Europe that were converted from forest to grassland, eventually transitioning back to forest. The time series plots show all Landsat observations (points) in the Landsat SWIR1 band, and the CCDC model fits (lines). The high-resolution images illustrate the land cover change on the ground. Map data: Google, Maxar Technologies.

GLanCE training data were collected by a team of trained image analysts at Boston University using the land cover key and a suite of online tools (https://github.com/parevalo/measures_collector, using Google Earth Engine API). Image analysts interpreted land cover on-screen using a combination of high-resolution Google Earth imagery, Landsat imagery, time series of spectral reflectance, vegetation indices, and Landsat-derived Tasseled Cap transformations. In addition, image analysts used Google Earth photos and StreetView (where available) to aid their interpretations. Hereafter we refer to each entry in the database, which represents individual Landsat pixels, as a training unit. Each training unit corresponds to an interpretation by an image analyst of Continuous Change Detection and Classification (CCDC) (https://developers.google.com/earth-engine/datasets/catalog/GOOGLE_GLOBAL_CCDC_V1) time segments (explained below) (Fig. 1). Each unit was assessed for quality and potentially flagged for review by a second image analyst. If one image analyst disagreed with another on the land cover label of a given training unit, a third team member reviewed and, if necessary, re-interpreted or removed the unit. Units were removed if there were no high-resolution imagery available and team members had no way of determining the land cover with high confidence.

Over the course of data collection, the team of image analysts consisted of 6 to 12 members who were trained to interpret satellite imagery for land cover attributes. Analysts alternated between interpreting sets of randomly assigned training units and reviewing peers’ interpretations. All image analysts received the same training to ensure consistency in their interpretation, followed by a practice training set for each continent that was collectively discussed so that analysts learned from their errors and followed consistent interpretation protocols. Training included image interpretation, guidance on how to use software tools that were developed to support this activity^40^ (https://github.com/parevalo/measures_collector), use of ancillary data (Table 1), and class definitions (Table 2). In addition, quarterly refresher trainings and weekly meetings were conducted to provide analysts with feedback regarding errors and inconsistencies in interpretations discovered during the review process. As a final step, all training data were reviewed for clerical errors and compiled into unique databases for each continent.

**Table 1:** Summary of data sources used to compile the GLanCE training database.

Dataset	Spatial extent	Years	Number of units	Original source
STEP	Global	1990–2020*	26,918	GLanCE^18^
CLUSTERING	Global	1990–2020*	33,385	GLanCE
Feedback	Global	1990–2020	23,271	GLanCE
GeoWiki	Global	2000–2012	11,833	^17^
GLC30	Global	2015	25,632	^19^
Training augment	Global	2015	14,080	^37,38^
MODIS-algo	Global	2018	4,583	^36^
LandCoverNet (Radiant Earth)	Africa, Asia, Australia	2018	73,469	^14^
ABoVE	Canada, Alaska	1984–2014*	6,547	^34^
LCMAP	United States	1984–2018	16,939	^20^
MapBiomas	South America	1985–2020	800,150	^27^
LUCAS	Europe	2009, 2012, 2015, 2018	587,753	^16^
ASB crop	Aral Sea Basin	2008, 2011, 2015, 2016, 2017, 2018	6,202	^25^
BU team collected	South America	1999–2019	17,471	^32,33^
	Colombia	2001–2017*		^31^
	West Africa	2001–2020*		GLanCE
	Georgia	2000*		^29^
	Laos	2017*, 2019*		^30^
Collaborator data	Ethiopia	2018	226,762	Unpublished
	Zambia	2008		Unpublished
	Ghana	2017		^28^
	Australia	1985–2019		^26^

STEP stands for System for Terrestrial Ecosystem Parameterization; ABoVE stands for Arctic Boreal Vulnerability Experiment; LCMAP stands for Land Change Monitoring, Assessment, and Projection; LUCAS stands for Land Use and Coverage Area frame Survey; GLC30 stands for Global Land Cover product with Fine Classification System; ASB stands for Aral Sea Basin.

^*^Collected to align with CCDC model fits.

**Table 2:** GLanCE Level 1 and 2 land cover legend definitions.

Level 1	Level 2	Description
Water (1)	Water (1)	Areas covered with water throughout the year: streams, canals, lakes, reservoirs, oceans.
Ice/snow (2)	Ice/snow (2)	Land areas with snow and ice cover greater than 50% throughout the year.
Developed (3)	Developed (3)	Areas of intensive use; land covered with structures, including any land functionally related to developed/built-up activity.
Barren/sparsely vegetated (4)		Land comprised of natural occurrences of soils, sand, or rocks where less than 10% of the area is vegetated.
	Soil (4)	Land covered with less than 10% vegetation and dominated by soil.
	Rock (5)	Land covered with less than 10% vegetation and dominated by rocks.
	Beach/sand (6)	Land covered with less than 10% vegetation and dominated by beach/sand.
Trees (5)		Land where tree cover is greater than 30%. Note that cleared trees (i.e., clear-cuts) are mapped according to *current* cover (e.g., barren/sparsely vegetated, shrubs, or herbaceous).
	Deciduous (7)	Land with tree cover greater than 30% and all trees present are deciduous.
	Evergreen (8)	Land with tree cover greater than 30% and all trees present are evergreen.
	Mixed (9)	Land with tree cover greater than 30% and neither deciduous nor evergreen trees dominate.
Shrub (6)	Shrub (10)	Land with less than 30% tree cover, where total vegetation cover exceeds 10% and shrub cover is greater than 10%.
Herbaceous (7)		Land covered by herbaceous plants. Total vegetation cover exceeds 10%, tree cover is less than 30%, and shrubs comprise less than 10% of the area.
	Grassland (11)	Herbaceous land covered with grass.
	Agriculture (12)	Herbaceous land covered with cultivated cropland.
	Moss/lichen (13)	Herbaceous land covered with lichen and/or moss.

The integer values assigned to land cover classes are indicated in parentheses for Level 1 and Level 2 labels.

Because a single Landsat pixel can include multiple land cover types over time, an important component of our training data collection protocol was the interpretation of land cover change. The core algorithm used in this project, the CCDC algorithm^41^, operates on time series of satellite data to identify abrupt and transitional changes, and stable land cover. The concept of a time segment—the period for which the intra- and inter-annual patterns of surface reflectance can be described by a single model fit—is central to the CCDC algorithm. To illustrate this concept, Fig. 1 shows a time series of shortwave infrared reflectance values for two Landsat pixels that both correspond to forest at the beginning of their time series, which then transition to grasslands and slowly evolve back to forests at the end of the time series. To confirm that various types of land-cover change are represented in the training database, changes such as those exemplified in Fig. 1 were explicitly included in the dataset and labeled according to the type of change (as described above). In the examples shown in Fig. 1, CCDC identifies distinct time segments between detected change events on the land surface (shown in different colors); each segment is assigned a single land cover label. The time segments representing stable land cover correspond to the subsets of Landsat time series with relatively constant spectral and temporal reflectance patterns over several consecutive years. In Fig. 1a, the forest was represented by a stable segment from 2000 to 2009. In contrast, transitional segments correspond to land cover that gradually transforms over the time, where consecutive annual patterns of surface reflectance change in a constant manner. For example, in Fig. 1b, forest regrowth from 2011 to 2019 is evident in the Landsat time series and identified by CCDC in the segment starting in 2011. Abrupt changes, such as the logging event in 2009 in Fig. 1a, corresponded to high magnitude breaks in the CCDC time segments. In the training dataset, stable and transitional labels were assigned based on visual interpretation of Landsat time series, CCDC models, and reference high resolution imagery. We based our labels on the condition of the land cover and on the slope of the time series.

### Data collected by the GLanCE team

**Fig. 2:**
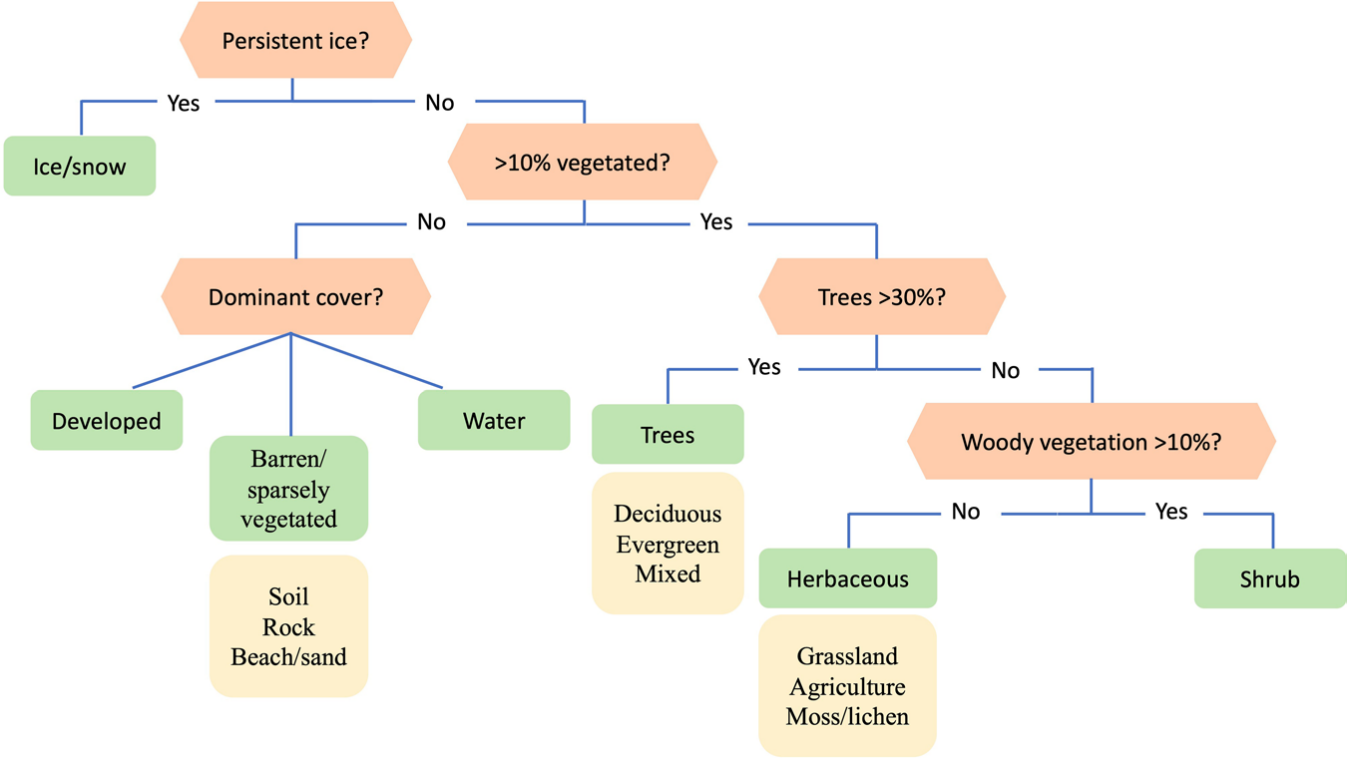
Training data key used to assign land cover attributes to training units. Green boxes show Level 1 land cover classes and yellow boxes show Level 2 classes.

Image analysts interpreted training units from three sources: (1) the System for Terrestrial Ecosystem Parameterization (STEP) training database^18^, (2) a sample generated via unsupervised clustering of Landsat spectral-temporal features, and (3) a sample of feedback training units generated to improve the accuracy of land cover classes or regions that were persistently misclassified. The STEP database, which was created to provide a representative sample of land cover from all global ecoregions, was created to support the MODIS Collection 6 Land Cover Type Product (MCD12Q1)^18^. To adapt the STEP database for GLanCE, we randomly selected 10 Landsat pixels within each MCD12Q1 500 m pixel and visually interpreted them using the procedure outlined in above and labeled them using the GLanCE land cover key (Fig. 2, Table 2). Only training units representing homogenous land cover were collected as training data; training units containing mixed land covers were removed.

The second set of training data that image analysts collected was based on unsupervised clustering of spectral-temporal features estimated from Landsat image time series using the CCDC algorithm. This approach was stratified using the World Wildlife Fund (WWF) ecoregions^39^ to ensure that each ecoregion was adequately represented in the training database. While STEP-based training data were designed to capture homogeneous land cover, a key goal of cluster-based sampling was to locate and collect training units with more heterogeneous land cover composition. Including units that represent heterogeneous land cover in the training dataset has been shown to improve classification results and is an efficient way to collect training samples at continental-to-global scales because a relatively small number of training units captures the variability in land cover spectral properties from each ecoregion^3,4,6–8^. As such, the cluster-based training data represented variation in land cover composition, stability, and intra- and inter-class spectral variability at the regional scale.

Our cluster-based approach included two main steps: (1) principal component analysis (PCA) to reduce the dimensionality of the data; and (2) k-means clustering on the principal components (PCs) to identify the optimal partitioning of the training data. For each ecoregion, we selected a maximum of 10 PCs to capture at least 80% of the variance, although 99% of the variance was frequently captured in fewer PCs. We ran k-means clustering for a range of K values from 5 to 400 and for each value we calculated the sum of squared distances from each point to its assigned center to select the optimal (fewest) number of clusters that were well separated from one another. As a result, we selected 30 to 60 clusters – for a maximum of 500 training units - per ecoregion. The resulting dataset was representative of the distribution of land cover at the regional scale and included a mix of homogeneous and heterogeneous training units. Because the STEP- and cluster-based datasets were collected explicitly for the purposes of the GLanCE project, together they represent the most thematically detailed and complete data in our database, with up to 23 recorded attributes per training unit.

The third set of training data were generated to iteratively improve the accuracy of land cover maps. Despite our best efforts to represent all ecoregions in the training data, examples of some specialized and regionally relevant land uses (e.g., greenhouses in Spain and China, sparse orchards, and plantations in India etc.) were missing in our training database, which resulted in obvious errors in the map results. To ameliorate these issues, we collected “Feedback” training units for these locations around the globe using the interpretation tools described above.

### Supplementary data sources

Given the global scale of the GLanCE project and the lack of available high-resolution imagery in some parts of the world, the GLanCE-collected dataset described above was insufficient to capture the full range of geographic, temporal, and spectral heterogeneity in global land cover. To address this, we supplemented the GLanCE training database by leveraging existing publicly available, collaborator-contributed, and team-collected datasets that we harmonized and standardized to conform to the GLanCE land cover classification key. The sources and key features of these data are summarized in Table 1. As part of this process, we worked with collaborators and team members with regional expertise in land cover and land cover change to harmonize their land cover legend with the GLanCE legend. This consisted of cross walking collaborator-provided land cover legends and definitions to the GLanCE legend, clarifying definitions with collaborators, and eliminating unsuitable classes from the pool of potentially useful training units. Data labeled as “BU team collected” were compiled from different existing projects by team members but weren’t explicitly collected for the GLanCE project (Table 1). Note that several of these data sets are publicly available and peer-reviewed, and all datasets were extensively vetted for quality control as described below. While GLanCE-collected training units were collected based on CCDC segments, most supplementary data sources were not.

Unfortunately, even after the data collected by the GLanCE team were combined with the supplementary datasets described above, some land cover classes, especially rarer classes (e.g., developed, water, shrub), were underrepresented. To address this, we augmented the database with training data derived from the World Settlement Footprint product^37^, the Global Surface Water product^38^, and by implementing the algorithm developed by Zhang & Roy (2017)^36^ that uses the MODIS Land Cover Type Product^18^ to automatically select candidate training units at Landsat resolution. Briefly, this algorithm estimates the 20th, 50th, and 80th percentile for thirteen different Landsat variables (bands and band ratios) for a total of 39 predictors, calculates the metric centroid (a vector of 39 metric average values) of all 30 m pixels located within 500 m MODIS pixels, and then selects the 30 m Landsat pixel with the smallest absolute difference from the metric centroid. Since the World Settlement Footprint and Global Surface Water products are produced at spatial resolutions that are comparable to Landsat, we drew a random sample of training units for each ecoregion from each product in each continent. We used these units to ensure that the training data were representative of the underlying distribution of land cover, both regionally and globally, which is a common problem in machine learning-based land cover classification^6,42^.

### Pre-processing and harmonization of supplementary data sources

Pre-processing and harmonization of supplementary datasets consisted of four steps: (1) if available, each dataset was filtered based on interpreter confidence (highest) or consensus score (100% agreement among interpreters); (2) the land cover legend for each data set was harmonized with the GLanCE legend (crosswalk tables in Supplementary Information); (3) each dataset was compared against an existing land cover product (ESA World Cover^43^, Copernicus Global Land Cover Layers^44^, or the MODIS Land Cover Type product^18^ depending on the time period of the supplementary data source), and training units were discarded where they disagreed with the existing product; and (4) we visually inspected approximately 30% of each harmonized and cleaned supplementary dataset in Google Earth using high resolution imagery to evaluate the overall quality and remove mislabeled training units. Step 3 provided an automated way to eliminate or reduce the number of poor-quality training units by ensuring that supplementary land cover labels agreed with at least one other data source. Note that this approach is susceptible to errors of omission and commission in the existing map sources, but because we only retain labels where both data sets agree, we assume this strategy is conservative and leads to relatively few errors. Step 4 is designed to ensure the overall quality of the training dataset and enables us to iterate on the ingestion process if necessary. For example, Step 4 occasionally resulted in supplementary datasets being reviewed and reinterpreted by GLanCE image analysts. Details regarding legend harmonization for each data set are included in the Supplementary Information. Despite our best efforts to harmonize and clean the supplementary training datasets, a constraint to our approach is that we had limited control over the external dataset’s accuracy and consistency.

## Data Records

The GLanCE training dataset described in this paper is available from Source Cooperative^45^ under Creative Commons license CC-BY-4.0.

The GLanCE land cover training dataset includes two nested sets of classes: seven broad, mutually exclusive classes (Level 1) and nine secondary classes (Level 2) (Table 2, Fig. 2)^24^. The GLanCE land cover classification scheme is designed to focus primarily on land cover and is compatible with common land use categories for greenhouse gas inventory reporting^46^ land cover classification systems such as the IPCC and the FAO Land Cover Classification System (LCCS)^24,47^. Land cover labels were assigned for 30 m Landsat training units based on fractional cover thresholds (Fig. 2). To label each training unit, we followed the stepwise decision-making process shown in Fig. 2. In addition to Level 1 & 2 land cover and land use labels, the dataset includes eight additional attributes that provide complementary information related to land cover and land use properties (Table S1). Note that these additional attributes (e.g., LC_Confidence, Segment_Type) were collected only for GLanCE training units and as a result are available only for ~3% of the dataset. Each training unit’s land cover label corresponds to a specific Landsat training unit and time period between 1984 and 2020 (Table S1). The years assigned to each unit are inclusive. For example, if the training unit is labeled “Trees” from 2000 to 2020, then it was trees for the full calendar years of 2000 and 2020. IDs are assigned based on latitude and longitude, so units with duplicate location and the samet ID indicate units that experienced land cover abrupt or gradual change. However, each training unit has a unique ‘Glance_ID’. Table S1 includes a complete list of attributes and descriptions.

Because land cover is dynamic and can change due to natural or anthropogenic processes, GLanCE training units are characterized as either ‘stable’ or ‘transitional’ (Segment_Type in Table S1) based on time series of both high-resolution imagery and Landsat observations (detailed description in Methods). A “stable” unit is defined as a time segment with a single land cover and consistent annual patterns of spectral reflectance across the length of the segment. Conversely, a “transitional” unit is a time segment in which the land cover gradually changes over the time, where the transition is reflected in the annual patterns of surface reflectance. Transitional units included examples of forest decline or degradation (Fig. 1), fire, insect damage, urban development, flooding, regrowth (Fig. 1), riparian/water shift, drought, as well as unknown transitions. For transitional processes that slowly transform landscapes, such as vegetation regrowth during ecological succession, the land cover was recorded at both the beginning and end of the time segment (typically the first and last three years) when the Level 1 land cover attributes remain unchanged (Fig. 1).

**Fig. 3:**
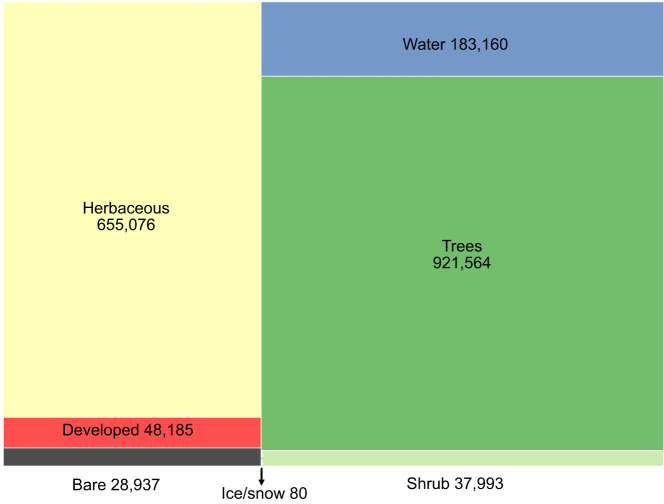
GLanCE global training units class distribution for Level 1 land cover. Note that the Bare label is shorthand for Barren/sparsely vegetated.

**Fig. 4:**
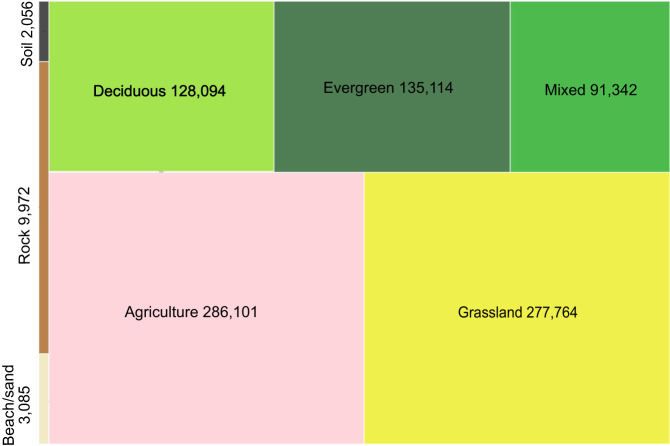
GLanCE global training data distribution for Level 2 land cover. Note that this figure doesn’t display the Moss/lichen category because it includes only 640 training units.

**Fig. 5:**
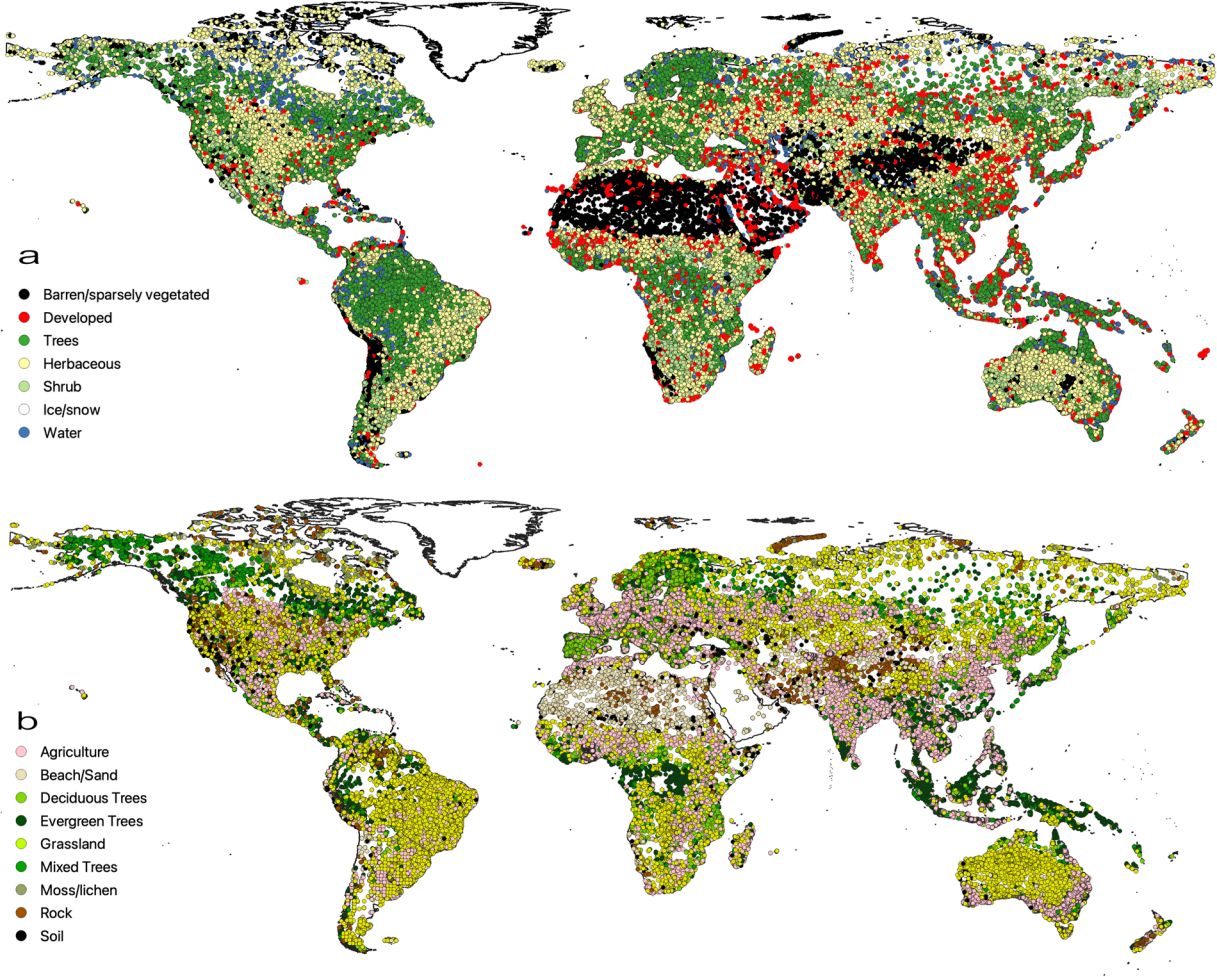
Global spatial distribution of GLanCE training units for Level 1 (**a**) and Level 2 (**b**) land cover. Note that the overall density of training units is lower in Africa and Asia, so the Developed class in (**a**) is visually overrepresented in this map.

The V1.0 training dataset consists of 1,874,995 training units distributed across the globe representing seven broad land cover classes at Level 1 and nine classes at Level 2. Approximately 79% and 21% of the dataset correspond to stable land cover and change, respectively. Note that the change category includes abrupt (land cover label change) and transitional processes such forest regrowth, coastal water dynamics etc. At global scale, change is a relatively rare occurrence but because it is inherently hard to map, change needs to be captured in the training database. Figures 3, 5a show the class frequency and geographic distribution of the training database for Level 1 land cover, while Figs. 4, 5b display the distribution for Level 2 land cover and land use. Level 1 training data are well distributed and representative of all ecoregions. However, only 50% of the training data contain Level 2 legend information (Figs. 4, 5). Despite our efforts to include Level 2 labels for supplementary datasets whenever possible, only half of the training units contain this information in the final training database because supplementary datasets often did not include land use (e.g., agriculture). Relatively rare classes on the landscape such as developed, barren/sparsely vegetated and shrubs are well-represented in the training database. In contrast, ice/snow is not well represented because it tends to be located in areas where Landsat data density is insufficient for CCDC (Fig. 3). The distribution of Level 2 labels is dominated by herbaceous classes such as grasslands and agriculture (Figs. 4, 5b). The detailed quality control procedures, described in the Methods section, were applied to Level 1 land cover labels only. Therefore, Level 2 labels and all other land cover attributes (e.g., leaf phenology) may not always meet the highest standards of quality and may need to be further filtered or processed to fit the user’s region and research of interest.

**Fig. 6:**
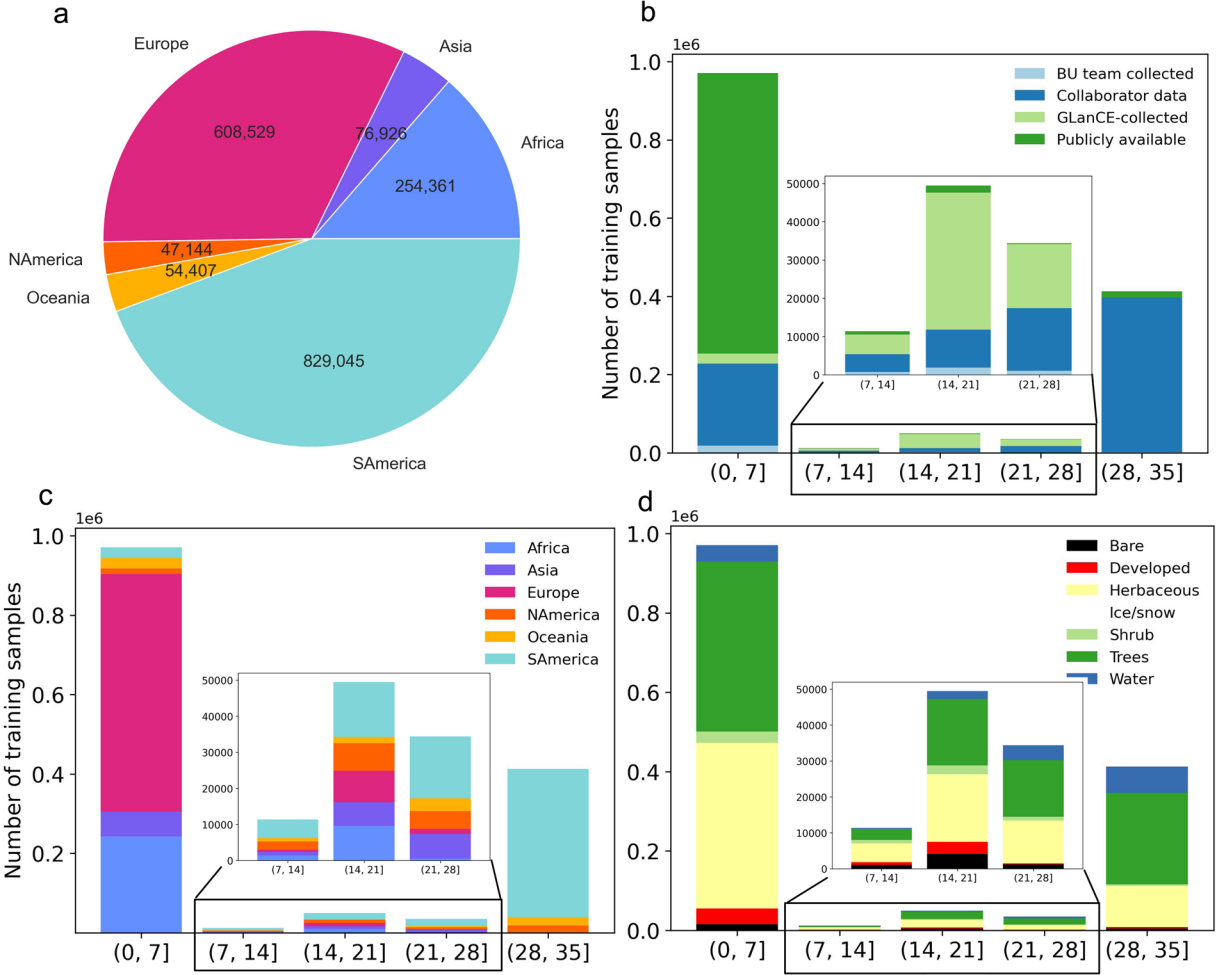
The distribution of training units across the different continents (**a**) and across time (**b**–**d**). Panels b thru d show the number of training units that belong to different bins of time segment lengths split by data source (**b**), continent (**c**), and land cover class (**d**). BU stands for Boston University. MODIS-derived units accounted for 0.2% of the total dataset so they are not shown in panel (**b**). SAmerica stands for South America and NAmerica stands for North America. The insets show more detail for the bins of time segment lengths with fewer observations.

To our knowledge, the dataset presented in this study is the longest, most extensive, and comprehensive publicly available global land cover and land use training database. We standardized and harmonized 22 disparate sources of land cover labels into a single unified training database that is comprised of 39% publicly available data, 55% collaborator-provided data, 4% GLanCE-collected data (collected explicitly for the purposes of the GLanCE product), 1% Boston University team collected data (collected by team members for other projects, not explicitly for the purposes of GLanCE), and 0.2% MODIS-derived training data (Table 1, Fig. 6b). Among the various data sets incorporated into the database, the GLanCE training data contain the highest level of detailed ancillary information on secondary land cover attributes and change information, and frequently span 20 years between 1999 and 2019 (Fig. 6b). Some collaborator-provided data sources such as MapBiomas, LCMAP, and ABoVE (Table 1) include up to 35 years of land cover labels and change information, while most publicly available data were limited to a single year (Fig. 6b). The training units are not equally distributed among continents (Fig. 6a) with Europe (source: LUCAS) and South America (source: MapBiomas) having the most data available. While training units in South America have time segment lengths of up to 35 years, most training units have time segment lengths of less than 7 years (Fig. 6c). Level 1 land cover classes are evenly distributed among all the time segment length bins (Fig. 6d).

## Technical Validation

Human error is inherent to all land cover training data sets, especially those compiled by on-screen interpretation^2^. However, the size of our database precludes manual quality assurance for each training unit. Hence, to minimize errors and maximize the dataset’s quality, we used a two-step machine learning-based cross-validation procedure adapted from Brodley & Friedl^48^ to remove poorly labeled training data. In the first step, we divided the training data in each continent into up to 9 biogeographical regions determined based on *k*-means clustering of maximum temperature, minimum temperature, precipitation, latitude, and longitude. Before clustering, the three climate variables were normalized to unit variance, and we included latitude and longitude to create spatially coherent clusters. In the second step, we used the ranger package in R to estimate unique random forest models for each cluster and to estimate class membership probabilities for each class for each training unit using a combination of remote sensing and ancillary features as predictors at each unit (see^24^ for details). The ancillary data features used to build random forest models include topography, global surface water, climate, population, and settlements.

We then examined the difference between the 1st and 2nd most likely classes; training data with margins less than 0.05 were discarded because they represented cases where the two most likely classes were easily confused. To select this threshold, we performed a 10-fold cross validation analysis, which demonstrated that training cases with margins less than or equal to 0.05 had substantially higher misclassification rates relative to cases with higher margins. We then removed all misclassified cases for which the margin between the predicted label and the label assigned to the unit in the database was in the upper quartile of margins for each class. In other words, we removed data where the assigned label differed from the label predicted by random forest, and where the class probability for the label assigned by random forest was high.

**Fig. 7:**
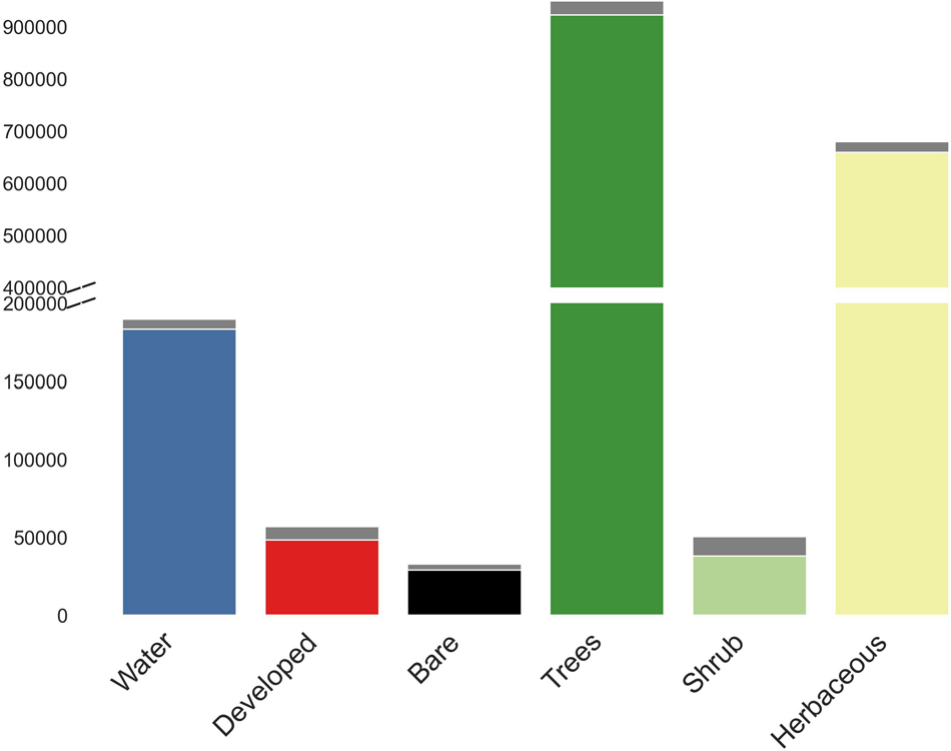
Class distribution before and after the filtering procedure. Gray bars show the removed units per class based on the cross-validation procedure. Note that the Bare label is shorthand for Barren/sparsely vegetated. The Ice/snow class contained only 214 units and is not included in this visual.

Using this procedure, we removed ~15% of the training data in each continent (Fig. 7 - removed data shown in gray). Approximately 97% of removed training units were discarded by applying the 0.05 threshold and 3% were discarded because the predicted and assigned label differed. The removal of training units was uniform across land covers (Fig. 7) and did not disproportionately affect transitional units (our procedure discarded 23% of all training units labeled transitional and 16% of all training units labeled stable). In some cases, training units with very small margins provide examples of land cover that are unique or represent important sub-variants of the classes we are mapping. In general, however, these cases represent training units with mixed land cover and the small margins associated with these training units indicate that the classifier is not able to reliably distinguish their land cover class, which can increase errors in classification results^48^. Hence, we adopted a strategy of removing these cases from the training set.

As an additional technical validation, we followed an approach used by Doda *et al*.^49^ to compare classification results based on our training data against reference data. We created the reference set by splitting our final training database into train (70%) and test (30%) data and withholding the test data from the model. We used the random forest classification algorithm in the scikit-learn package (version 0.22.2) in Python 3.6.7. We selected random forest because it is 1) a widely-used model suitable for land cover mapping, 2) relatively resistant to overfitting, and 3) efficiently handles noisy input data^50,51^. To optimize random forest, we used a grid search to automatically fine-tune model parameters and 3-fold cross-validation to assess model performance. To estimate the final model used in this technical validation, we used the parameter combination with the lowest root-mean-squared error (RMSE). To train the model, we used predictors derived from the CCDC parameters based on harmonic models fit to time series of Landsat surface reflectance bands (e.g., green phase, green amplitude, etc.) as well as a variety of ancillary layers (e.g., topography, population, etc.)^24^. In total, each training unit had 56 features.

**Fig. 8:**
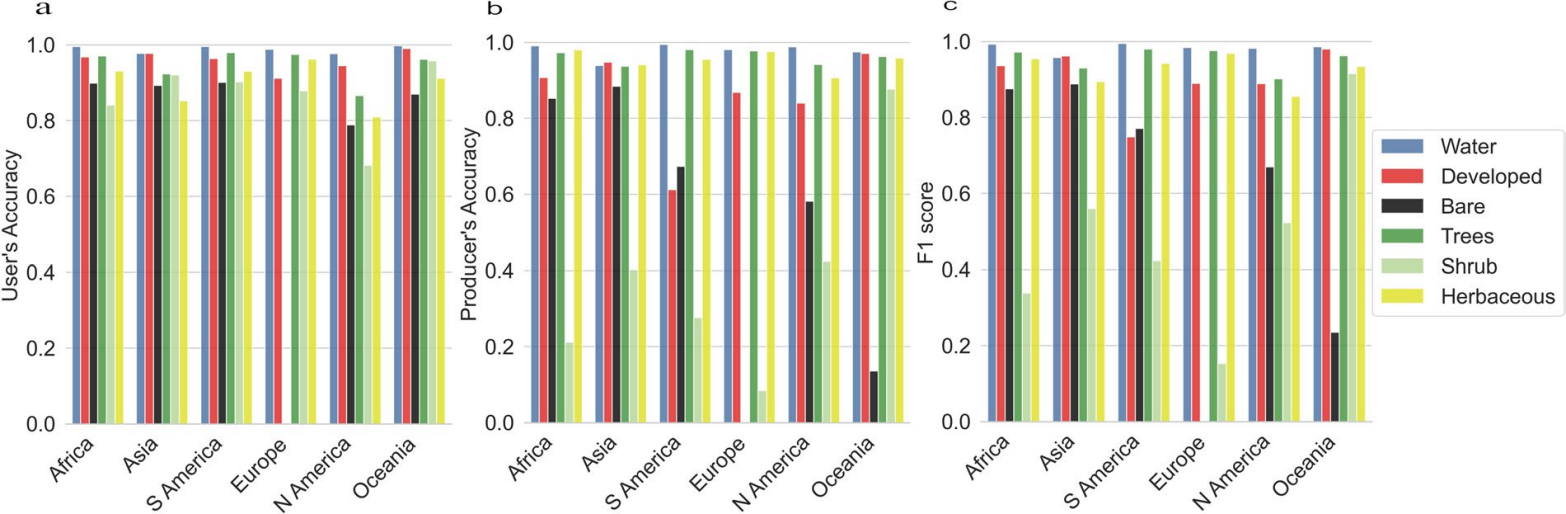
User’s accuracy (**a**), producer’s accuracy (**b**), and F1 score (**c**) for each continent and each land cover class (except Ice/Snow as there weren’t enough training units available for this class). N America stands for North America, and S America for South America.

Tables S2–S7 show the confusion matrices between the observed and predicted land cover labels. Even though the user’s accuracy is high (greater than 0.8 in most continents) (Fig. 8a), there is confusion between some classes (Tables S2–S7). Distinguishing between herbaceous vegetation and shrubs is difficult because frequently training units represent a mixture of these two cover types, an issue that has been well documented by previous studies^18,24^. Even at 30 m spatial resolution, relatively few training units are actually uniform in terms of their land cover composition. Note that producer’s accuracy and F1-score are generally high for most classes, but low for shrubs for almost every continent except for Oceania (Fig. 8b,c). Producer’s accuracy is also low for developed and bare ground, especially in South America (Fig. 8b).

## Usage Notes

Because the process of acquiring supplementary datasets was opportunistic and non-systematic based on data availability and quality, the full database includes geographic variation in data density. For example, some regions have training units that are geographically clumped (e.g., Ghana) or land cover classes that are overrepresented (e.g., herbaceous) (Figs. 3–5). Some users may need to sub-sample the dataset or enforce constraints on data density depending on their research question, application, or area of interest. For example, in the GLanCE project, we use a 100 × 100 km grid to assign weights such that if more training data are present in a single grid cell, the data are sub-sampled (and vice-versa) to ensure pseudo-uniform density of training data across space. Users may also need to sub- or resample the database to enforce uniform or proportional class distributions.

For applications focused on land cover change (abrupt or gradual), for which our database includes proportionally less data, we recommend retaining all change training data (for guidance see^3,11,13^). For applications focused on agriculture, users can use the Level 2 category of ‘Agriculture’ as a starting point but should be aware that this label has not undergone rigorous quality control and is available for a limited subset of the global training dataset (286,284 units). Users should apply quality control measures, depending on their applications, such as filtering Level 2 attributes as outlined in the Technical Validation, intersecting the agriculture units with existing regional cropland maps as outlined in Methods, and visual inspection of training units in Google Earth.

### Supplementary information

Supplementary_Information

Supplementary_Information_crosswalk_tables

## Data Availability

We used open-source tools to ensure transparency and reproducibility of our research, including R (4.3.0), Python 3.6.7, and Google Earth Engine. Time series tools for training data collection are available on GitHub (https://github.com/parevalo/measures_collector) as is the repository for filtering training data (https://github.com/ma-friedl/GlanceFiltering). Custom continental definitions can be found at this repository: https://measures-glance.github.io/glance-grids/params. Continuous Change Detection and Classification (CCDC) tools and applications can be found on Google Earth Engine (https://glance.earthengine.app/view/fulltstools) and python (https://github.com/repository-preservation/lcmap-pyccd).
