# Supplementary material for: A global land cover training dataset from 1984 to 2020
Source: Sci Data. 2023 Dec 7;10:879. doi: 10.1038/s41597-023-02798-5 (PMC10703991; doi:10.1038/s41597-023-02798-5)
Supplement: Supplementary file 1 — Supplementary_Information [file 41597_2023_2798_MOESM1_ESM.pdf]

Table of Contents

Table S1 2

Table S2 3

Table S3 3

Table S4 4

Table S5 4

Table S6 5

Table S7 5

Table S1. Full list of training unit attributes and descriptions.

Column Name	Description
Lat	Latitude
Lon	Longitude
Start_Year	Start year of segment, ranging from 1984 to 2020 (integer)
End_Year	End year of segment, ranging from 1984 to 2020 (integer)
Glance_Class_ID_level1	Level 1 land cover value (integer): 1 (Water), 2 (Ice/snow), 3 (Developed), 4 (Barren/sparsely vegetated), 5 (Trees), 6 (Shrub), and 7 (Herbaceous). See Table 1 for detailed class definitions.
Glance_Class_ID_level2	Level 2 land cover value (integer): 1 (Water), 2 (Ice/snow), 3 (Developed), 4 (Soil), 5 (Rock), 6 (Beach/sand), 7 (Deciduous), 8 (Evergreen), 9 (Mixed), 10 (Shrub), 11 (Grassland), 12 (Agriculture), and 13 (Moss/lichen). Value of 0 is no data available.
Leaf_Type	Tree leaf type: broadleaf (1), needleleaf (2), and mixed (3). NaN values present.
Impervious_Percent	Impervious percent for developed training units: low 0%-30% (1), medium 30%-60% (2), and high 60%-100% (3). NaN values present.
Tree_Location	Binary integer indicating whether trees are on the interior (0) or edge (1) of a forest. NaN values present.
Veg_Density	Vegetation density for trees and shrubs: sparse 0%-30% (1), open 30%-60% (2), and closed 60%-100% (3). NaN values present.
Veg_Modifier	Vegetation modifiers, which can include one or more of the following: Cropland, Plantation, Wetland, Riparian/Flood, Mangrove, Greenhouse, and Trees/Shrub Present. NaN values present.
Segment_Type	Indicates whether a segment is stable (0) or transitional (1). See Section 1 for a detailed description. Land cover for transitional segments is recorded at both the beginning and end of the time segment - typically the first and last three years. NaN values present.
Change	Indicates presence (1) or absence (0) of land cover change for Level 1 land cover labels. Includes both abrupt change and gradual change (transitional segments (1) from the Segment_Type attribute) if it happened at any time for that training unit.
LC_Confidence	Interpreter confidence in the Level 1 land cover label from 1 (lowest) to 3 (highest). NaN values present.
Level1_Ecoregion	Ecoregion Level 1 number based on World Wildlife Fund definitions. For North America we used ecoregions based on the Environmental Protection Agency's Ecoregions of North America product (https://www.epa.gov/eco-research/ecoregions-north-america).
Level2_Ecoregion	Ecoregion Level 2 number based on the Environmental Protection Agency's Ecoregions of North America product. This field is available only for North America and is assigned a value of 0 for all other continents.
Continent_Code	Assigned continent number: North America (1), South America (2), Africa (3), Europe (4), Asia (5), and Oceania (6).
Dataset_Code	Assigned dataset number: 1, 2, 3, 4, 5, 902, 999, 700, 701, 702, 703, 704, 705, 706, and 707. Numbers correspond to each dataset as follows: STEP, CLUSTERING, LCMAP, ABoVE, MapBiomas, Feedback, Training_augment, MODIS_algo, GeoWiki, RadEarth, Collaborator_data, BU_team_collected, GLC30, LUCAS, ASB_crop. For details see Table 1.
Glance_ID	Unique ID for each training unit.
ID	ID for each unique combination of latitude and longitude. Change units have the same ID but different Glance_ID.

Table S2. Error matrix for GLanCE land cover training database for Africa. Note that the Bare label is shorthand for Barren/sparsely vegetated.

	Water	Developed	Bare	Trees	Shrub	Herbaceous
Water	7926	0	0	5	0	66
Developed	2	4310	30	43	2	364
Bare	20	22	2373	14	13	340
Trees	2	22	2	22314	11	588
Shrub	0	11	101	180	418	1265
Herbaceous	13	88	133	427	53	35188

Table S3. Error matrix for GLanCE land cover training database for Asia. Note that the Bare label is shorthand for Barren/sparsely vegetated.

	Water	Developed	Bare	Trees	Shrub	Herbaceous
Water	1224	3	0	13	0	63
Developed	2	4790	18	42	0	202
Bare	10	4	1972	5	1	238
Trees	0	36	0	8795	28	523
Shrub	0	5	44	342	698	646
Herbaceous	6	63	175	326	31	9662

Table S4. Error matrix for GLanCE land cover training database for Europe. Note that the Bare label is shorthand for Barren/sparsely vegetated.

	Water	Developed	Bare	Trees	Shrub	Herbaceous
Water	3146	1	0	41	0	19
Developed	0	2728	0	67	0	347
Bare	11	3	0	60	2	149
Trees	21	88	0	96183	4	2064
Shrub	0	4	0	638	94	384
Herbaceous	0	167	0	1690	7	74758

Table S5. Error matrix for GLanCE land cover training database for North America. Note that the Bare label is shorthand for Barren/sparsely vegetated.

	Water	Developed	Bare	Trees	Shrub	Herbaceous
Water	914	0	0	7	1	3
Developed	0	807	29	20	35	70
Bare	10	20	497	33	87	207
Trees	4	5	0	4129	74	173
Shrub	0	6	51	329	797	699
Herbaceous	8	16	53	253	175	4893

Table S6. Error matrix for GLanCE land cover training database for Oceania. Note that the Bare label is shorthand for Barren/sparsely vegetated.

	Water	Developed	Bare	Trees	Shrub	Herbaceous
Water	1434	0	3	9	0	25
Developed	0	5105	0	31	0	124
Bare	2	11	20	1	0	113
Trees	0	12	0	12751	84	393
Shrub	0	0	0	125	2758	262
Herbaceous	1	27	0	342	37	9456

Table S7. Error matrix for GLanCE land cover training database for South America. Note that the Bare label is shorthand for Barren/sparsely vegetated.

	Water	Developed	Bare	Trees	Shrub	Herbaceous
Water	40745	0	2	101	0	116
Developed	8	507	22	10	0	281
Bare	60	0	1661	28	5	712
Trees	55	0	0	138198	16	2584
Shrub	3	0	26	105	417	959
Herbaceous	40	19	132	2671	24	61766
